# Telemedicine/Telerehabilitation to Expand Enhanced Recovery After Surgery Interventions in Minimally Invasive Mitral Valve Surgery

**DOI:** 10.3390/jcm14030750

**Published:** 2025-01-24

**Authors:** Pietro Giorgio Malvindi, Maria Gabriella Ceravolo, Marianna Capecci, Stefania Balestra, Emanuela Cinì, Antonia Antoniello, Lucia Pepa, Antonella Carbonetti, Maurizio Ricci, Paolo Berretta, Francesca Mazzocca, Marco Fioretti, Umberto Volpe, Christopher Munch, Marco Di Eusanio

**Affiliations:** 1 Cardiac Surgery Unit, Azienda Ospedaliero Universitaria delle Marche, Polytechnic University of Marche, 60126 Ancona, Italy; 2 Neurorehabilitation Clinic, Department of Experimental and Clinical Medicine, Azienda Ospedaliero Universitaria delle Marche, Polytechnic University of Marche, 60126 Ancona, Italy; 3 Rehabilitation Unit, Azienda Ospedaliero Universitaria delle Marche, 60126 Ancona, Italyemanuela.cini@ospedaliriuniti.marche.it (E.C.);; 4 Department of Experimental and Clinical Medicine, Polytechnic University of Marche, 60126 Ancona, Italy; a.antoniello@outlook.it; 5 Department of Information Engineering, Polytechnic University of Marche, 60121 Ancona, Italy; 6 Unit of Clinical Psychiatry, Department of Clinical Neurosciences, Azienda Ospedaliero Universitaria delle Marche, Polytechnic University of Marche, 60126 Ancona, Italy; 7 Cardiac Anaesthesia and Intensive Care Unit, Azienda Ospedaliero Universitaria delle Marche, 60126 Ancona, Italy

**Keywords:** ERAS, telemedicine, mitral valve, minimally invasive surgery, cardiac surgery

## Abstract

**Objectives**: Having achieved a consolidated in-hospital experience with enhanced recovery after cardiac surgery, we explored the feasibility of expanding our protocol to pre-admission and post-discharge periods. **Methods**: A multidisciplinary team including cardiac surgeons, anaesthetists/intensivists, physiatrists, physiotherapists, perfusionists, nurses, psychiatrists, and engineers, elaborated a new therapeutic offer, based on current ERAS evidence and using telerehabilitation, to enhance preoperative communication and education and improve pre- and postoperative health and psychological state. **Results**: An institutional web-based platform for remote rehabilitation will host digital content that covers three main areas, including information and communication, prehabilitation and rehabilitation with the offer of respiratory and muscular exercises and aerobic activities, and psychological and patient experience evaluations. These interventions will be achieved through purposely developed video tutorials that present the hospital environments, the relevant healthcare personnel, and their role during the in-hospital patient’s journey, and illustrate tailored prehabilitation activities. A series of questionnaires will be administered to evaluate and follow the patient’s psychological state and collect patient-reported experience measures. The platform was activated in September 2024 and this service will initially involve 100 patients undergoing minimally invasive mitral valve surgery. A first review of compliance and engagement will be carried out after four months and a complete review of the results after the first year. **Conclusions**: ERAS is associated with improved surgical outcomes. A person-centred treatment should also address the health and psychological difficulties that patients face before hospitalisation and after discharge. Telemedicine is a valid tool to expand treatment and monitoring outside the hospital. This experience may give new insights into the feasibility and effectiveness of providing home-based remote interventions aimed at a global improvement in results throughout the overall cardiac surgery journey.

## 1. Introduction

Heart valve operations are nowadays associated with a low rate of postoperative mortality and morbidity [1,2] and provide an excellent durability of technical results [3] and improved survival probabilities [4]. Despite these achievements, surgery can affect, although transiently, the quality of life and functional capacity, especially in the first postoperative period.

The Enhanced Recovery After Surgery (ERAS) approach was developed to address these issues to improve surgical outcomes, ameliorate patients’ and operators’ experience, and reduce costs. After acquiring solid evidence in oncology, breast, and thoracic surgery [5], ERAS was finally integrated into cardiac surgery with the recent publication of the first dedicated guidelines and consensus statements [6,7,8]. ERAS’ strength lies in the aggregation of marginal gains that provide cumulative and meaningful improvements in both surgical outcomes and patient satisfaction. Based on this concept, over the last 8 years, we have developed a multidisciplinary approach for our practice in minimally invasive cardiac surgery, which has resulted in the progressive recognition, adoption, and amelioration of several key actions in the preoperative, intraoperative, and postoperative phases of the surgical journey. Having achieved a shared team experience in providing this enhanced in-hospital management [9], we decided to expand our ERAS approach by introducing the aid of telerehabilitation. The World Health Organization (WHO) defines telemedicine in a broader sense as “the delivery of healthcare services, where distance is a critical factor, by all healthcare professionals using information and communication technologies for the exchange of valid information for diagnosis, treatment and prevention of disease and injuries, research and evaluation, and for the continuing education of healthcare providers, all in the interests of advancing the health of individuals and their communities”. Under the umbrella term of digital health, telerehabilitation is defined as “the delivery of medical or rehabilitative care to persons with rehabilitation needs via telecommunication or the internet” [10]. By releasing the guidelines on digitally offered interventions, the WHO expressed its position in favour of digital technologies as tools with the potential to enhance the quality and coverage of health services. In line with the efforts of the WHO, telerehabilitation emerges as a priority for an ageing population with growing functioning problems leading to rehabilitation needs.

In this paper, we describe the context, rationale, and modalities of establishing these ERAS interventions, aiming for a more comprehensive treatment that starts before hospital admission and takes care of patients during their convalescence

## 2. Ethics

The information reported in this manuscript does not involve humans or include any patient data. Therefore, no ethics approval was required for this study.

## 3. Background

Since 2016, we have developed our practice in minimally invasive cardiac surgery within a multidisciplinary approach involving anaesthesiologists/intensivists, perfusionists, and physiotherapists. This led to the 360° minimally invasive cardiac surgery concept that incorporated different actions by each of these professionals with the aim of improving the whole surgical process and patient recovery. Cornerstones of this practice were as follows:

- A reduction in tissue trauma by performing cardiac surgery through mini-sternotomy access for aortic valve and aortic procedures and right mini-thoracotomy trans-axillary access mainly for atrioventricular valve pathologies. These two approaches were characterised by the possibility of performing all the surgical procedures through direct vision, without the need for any special tool (i.e., camera, endo-aortic balloon occlusion system) with operative times at least similar to those registered when operating through conventional full sternotomy [11,12,13];
- Respect for physiology, thus favouring cardiopulmonary bypass in normothermia with extensive use of minimally invasive (Type IV MiECC) [14] or optimised extracorporeal circulation systems;
- Low-dose opioid anaesthesia and aggressive pain control with loco-regional chest wall analgesia to achieve on-table extubation, or within 6 h since the end of the procedure;
- Physiotherapy starting within 3–6 h of admission to the intensive care unit with bed mobilisation and chest therapy. Standing position exercise on day 1 with prompt re-establishment of oral feeding and drains and lines’ removal.

These key points were progressively codified into our “Ultra-fast track” protocol, which has been extensively discussed and presented in previous publications (Supplemental Table S1) and was found to be associated with shorter mechanical ventilation time, shorter ICU hospital stays, and a higher rate of discharge straight home without further rehabilitation [9,15].

## 4. Elaboration of an Expanded ERAS Protocol

A multidisciplinary team including cardiac surgeons, anaesthesiologists/intensivists, physiatrists, physiotherapists, psychiatrists, theatre/ICU and ward nurses, and perfusionists was established at the end of 2023 to review the results associated with our “Ultra-fast track” in-hospital protocols and discuss the opportunity to expand our ERAS pathway by adding further key actions in the pre-admission and post-discharge phases. The team reviewed the literature on existing ERAS protocols in heart valve surgery to evaluate the organisation behind these interventions and the modalities of their implementation as previously proposed by leading groups in the field of cardiac ERAS [16].

During the first meeting, a general consensus was reached on the use of telerehabilitation as the most convenient way to offer information, education, and treatment to patients before hospital admission and to provide, alongside the usual outpatient clinic activity, a punctual follow-up during the postoperative period. A request was made to activate an area dedicated to this project on the institutional K-Rehab website [17]. This telerehabilitation platform was developed in collaboration between the Department of Clinical Experimental Medicine—Neurorehabilitation section and the Department of Information Engineering of the Polytechnic University of Marche within the framework of funded (GR GR-2011-02349761, Cariverona-funded project RAPIDO = teleRehAbilitation for PatIents with parkinsons’s Disease Of any stages) or spontaneous research projects, and in 2021 within the RAPIDO project it acquired the collaboration of REVOLT s.r.l.. The platform was registered with the Italian Ministry of Health as a Level I medical device. In 2020, the University in collaboration with the University Hospital of Marche, Neurorehabilitation Clinic, made the platform available to guarantee a remote physiotherapy service for patients with rehabilitation needs due to orthopaedic, respiratory, or neurological pathologies, in order to overcome the limitations caused by the COVID-19 pandemic. During the second meeting, the team evaluated the contents to be offered through the web-based platform and discussed with an academic engineer from our university the technical feasibility of providing video tutorial material and psychological tests and the possibility of anonymous data management. The third and the fourth meetings ultimately defined the modalities of delivery of the contents, particularly regarding the need to tailor this offer according to different pathologies and patients’ clinical status.

The adoption of ERAS-based interventions during both the pre-admission and post-discharge periods aims to improve surgical outcomes and the patient’s experience throughout the surgical journey. It also contributes to build or reinforce the trust relationship between patients and care givers. Attention was given to patients’ emotional and psychological states to diagnose and treat any preoperative anxiety and depression, improve the physical fitness of patients while on the waiting list, and train them in exercises and physiotherapy activities that patients will be required to perform during the postoperative period. The completeness of this project’s presentation was checked following TIDieR recommendations (Supplemental Table S2) [18].

### 4.1. Design of the Web-Based Platform

**Figure 1:**
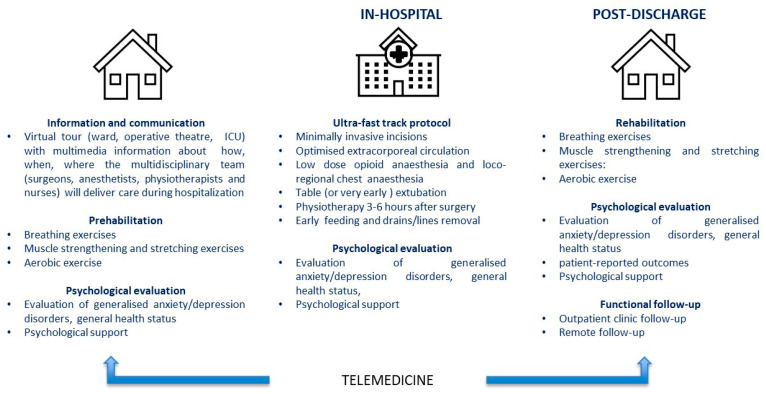
Architecture of the platform.

The online platform homepage presents three links to preadmission, in-hospital, and post-discharge treatment. The video abstract (Video S1 can be found in Supplemental Materials) and Figure 1 display the architecture of the platform and the list of the contents.

The preadmission area includes three fields:

- Information and communication;
- Prehabilitation;
- Psychological evaluation.

Similarly, the post discharge area gives access to the following:

- Rehabilitation;
- Psychological evaluation;
- Functional follow-up.

The in-hospital area hosts the psychological evaluation test, which will be undertaken during hospitalisation.

### 4.2. Information and Communication

Communication is enhanced by video tutorials displaying (a) a virtual tour of the hospital environments that will host patients during their stay—cardiac surgery ward, the transfer area to the theatre, the theatre, the intensive care unit, and the high dependency unit; (b) the cardiac surgeon informing about the underlying pathology, rationale, and expectations from the surgical treatment; (c) the anaesthesiologist describing the role of the anaesthesia and the pain management; (d) the perfusionist presenting the heart–lung machine and the concept of cardiopulmonary bypass b; (e) specialist nurses illustrating the course in theatre, ICU, and regular ward; (f) the physiotherapists explaining the engagement in physiotherapy and recovery expectation.

### 4.3. Prehabilitation

The team recognised two main goals of prehabilitation: increasing patients’ fitness while waiting for the cardiac operation (improving physical performance) and educating patients on how to perform postoperative exercises and activities (increasing compliance and reducing stress).

Each patient will receive access to a tailored exercise programme (basic, intermediate, advanced) based on the evaluation of their clinical profile (symptoms and heart function) and frailty score (in the absence of cognitive deficits and physical limitations that could preclude rehabilitation) at the time of enrolment (Table 1).

The exercises included in the prehabilitation programme are inspired by the so-called “Otago Exercises” [19], originally designed as home-based exercises aimed at preventing falls in more vulnerable patients.

This type of activity was chosen because it can be performed independently and does not require particular physical abilities or specific equipment. Furthermore, due to the nature of the telerehabilitation project, it can ensure a high level of safety for patients with cardiovascular pathologies and allows, with appropriate modifications to the number and complexity of exercises, for the treatment of patients with different clinical and frailty profiles.

The proposed exercises are categorised into four groups:

- Strengthening exercises;
- Stretching exercises;
- Aerobic exercises;
- Breathing exercises.

***Strengthening and stretching exercises*** target the major muscle groups of the trunk, lower limbs, and upper limbs.

In all training levels, the muscle groups involved are as follows:

- Trunk extensors;
- Ankle extensors and flexors;
- Leg extensors and flexors;
- Upper-limb extensors and flexors;
- Arm adductors and abductors.

The specific muscles trained include latissimus dorsi, trapezius, quadriceps, biceps femoris, triceps surae, dorsal foot extensors, biceps brachii, and triceps brachii.

According to patients’ characteristics, the prehabilitation programme includes the following:

Basic level: Lateral trunk flexions, head rotations, arm flexion–extension, arm adduction–abduction, leg flexion–extension, ankle flexion–extension + calf muscle (triceps surae) stretching, and arm muscle stretching (seated);

Intermediate level: In addition to Basic level exercises, leg adduction–abduction, toe–heel exercises, isometric arm exercises, quadriceps stretching, and additional arm stretches (standing);

Advanced level: In addition to Intermediate level exercises, mini squats, sitting and standing from a chair, marching in place + stretching similar to the intermediate level.

Every 4–5 exercises, the Borg Scale is introduced for self-assessment of fatigue, advising patients that if the score exceeds 6 to rest until fully recovered.

***Aerobic exercise*** includes interval walking training:

Basic level: 3 min of walking and 1 min of rest, repeated 4 times;

Intermediate level: 5 min of walking and 1 min of rest, repeated 4 times;

Advanced level: 10 min of moderate-intensity walking and 1 min of rest, repeated 3 times.

***Breathing exercises*** are the same for all three groups and consist of controlled breathing exercises aimed at improving awareness of the breathing pattern, deep breaths, and PEP (positive expiratory pressure) exercises with partially closed lips. Additionally, patients are instructed on how to cough correctly.

**Table 1:** Prehabilitation exercise protocols according to preoperative patients’ characteristics.

	Basic	Intermediate	Advanced
Clinical criteria	Symptomatic (NYHA II-III) and LV dysfunction Pulmonary hypertension Ventricular arrhythmia	Symptomatic (NYHA II) or LV dysfunction Pulmonary hypertension	Pauci/asymptomatic (NYHA I) and NO LV dysfunction NO pulmonary hypertension
CFS score [20]	4–6	3	1–2
Respiratory exercises	5 min twice a day three times a week	5 min twice a day three times a week	5 min twice a day three times a week
Muscle exercises	15 min of strengthening three times a week	20 min of stretching and strengthening three times a week	25 min of stretching and strengthening three times a week
Aerobic exercises	12 min three times a week	20 min three times a week	30 min three times a week

CFS: Clinical Frailty Scale. LV (left ventricular) dysfunction: LVEF < 60%, LVEDD < 60 mm. Pulmonary hypertension: pulmonary artery systolic pressure >50 mmHg at rest.

A similar training design will be provided for home rehabilitation after discharge. In this case, each patient will be able to sustain the appropriate level of exercise according to their clinical and functional status as evaluated by cardiac surgeons and physiotherapists at the time of hospital discharge.

### 4.4. Psychological Evaluation and Registration of Patient-Reported Experience and Outcomes

Preoperative psychological evaluation will be undertaken using online questionnaires incorporated into the web platform and exploring the measures of generalised anxiety disorder (GAD-7), the general health status (GHQ-12), and the depression status (PHQ-9). Based on the results of these questionnaires, when necessary, personalised psychological support can be provided before and during the hospitalisation via remote or in-person meetings. These tests will be repeated during the hospital stay and the first three weeks after discharge in adjunct to the evaluation of post-traumatic stress disorder (IES-6), the World Health Organization Disability Assessment Schedule 2.0 (WHODAS 2.0), and the administration of Visual Analogic Scales exploring patients’ satisfaction, the degree of pain and well-being, and the recovery of mobility and autonomy.

### 4.5. Population

The platform was activated in September 2024. This service will be initially offered to 100 patients undergoing elective mitral valve operation in isolation or associated with tricuspid and atrial fibrillation surgery. These patients usually spend 4 to 6 weeks on our waiting list, and this period was deemed adequate to implement all the preadmission actions. The possibility of entering the expanded ERAS protocol will be presented to patients during the outpatient clinic led by the cardiac surgeon with a tailored login profile according to the patient’s clinical status and characteristics, and a contact for any technical queries will be provided.

A first evaluation of this service will be carried out after four months to study patients’ compliance, their involvement in the proposed activity, and their effective use of the web platform. The second comprehensive evaluation will be performed 12 months after the start of the protocol.

## 5. Comment

Enhanced Recovery After Surgery (ERAS) is a “multimodal, transdisciplinary care improvement initiative to promote recovery of patients undergoing surgery throughout their entire perioperative journey” in order “to reduce complications and promote an earlier return to normal activities” [6]. This concept provides the adoption and/or the amelioration of several key actions in the management of the surgical patients, which are synergistically expected to lead to a significant improvement in surgical outcomes, patients and operators’ experience, and reduction in costs. These interventions, as widely reported in various publications by the ERAS study groups, usually include the enhancement of preoperative patients’ health status, physical, and psychological conditions; the integration of technical and technological surgical solutions aimed at a reduction in tissue and biological trauma; the improvement of pain control; reduction in mechanical ventilation time; and the possibility of an early mobilisation to allow for a faster recovery. Although well-established in general and thoracic surgery, the ERAS approach has only recently been adopted in cardiac surgery. Within a great heterogeneity in the proposed protocols, experiences in heart valve surgery have consistently reported similar mortality and postoperative cerebral stroke rates but lower mechanical ventilation time, ICU stay, and hospitalisation, when comparing outcomes of patients treated following an ERAS pathway with those obtained with conventional surgical management. Reduced costs and lower use of opioids were also found to be associated with an enhanced recovery treatment [16].

Starting in the late 90s, several experiences have shown that minimally invasive cardiac surgery can provide outstanding and durable surgical results [21,22]. However, its low penetration in real-world practice [23,24,25,26] and the few recommendations supporting its role in ameliorating patients’ outcomes [6,7,8] suggest that for many operators, minimally invasive surgery remains a mere technical commitment in performing cardiac operations by replicating conventional surgery through a reduced chest wall access, which requires a further learning curve and introduces the burden of longer operative times.

This view must be overcome, as the current standards in cardiac surgery have demonstrated that minimally invasive operations can facilitate the adoption of an ERAS protocol which can amplify the benefits of surgery through reduced chest wall incisions [9,27]. Based on this belief, we have developed and progressively refined our practice in minimally invasive cardiac surgery by integrating several actions aimed at reducing tissue trauma by providing smaller chest wall access and setting up surgical techniques that do not increase the complexity of the operations and avoid prolonged circulatory and cross-clamp times, thus respecting the physiology and containing the biological trauma. In this regard, perfusionists were called to optimise the CPB conduct and anaesthetists to provide fast-track anaesthesia to allow for physiotherapy to be started a few hours after the end of the surgical procedure. The complete description of the key actions included in our Ultra-fast track protocol and the results of this practice can be found in our previous publications [9,13,15].

Having consolidated this enhanced in-hospital management path, we have further embraced the spirit of the ERAS concept and decided to expand our offer through telemedicine in order to improve the pre-admission and post-discharge periods. As reported in the Section 4, this opportunity was discussed and then finalised within a multidisciplinary team after a careful review of the cardiac ERAS guidelines [6,7,8] and the study of several protocols and previously published experiences [16]. Each of the figures involved was called upon to provide specialist recommendations and solutions, which were finally evaluated by the whole team, also considering their potential effectiveness, patient safety, and their applicability on the web-based platform. For this last aspect, the role of a specialised engineer was fundamental in realising a simple and intuitive web user interface.

While digital health represents a global strategy of the WHO to improve health for everyone everywhere by accelerating the development and adoption of appropriate, accessible, affordable, scalable, and sustainable person-centric digital health solutions, the implementation of telemedicine in cardiac surgery is recent as it was undoubtedly driven by the COVID-19 pandemic, which has led to a forced shift in practice in preoperative and postoperative care towards remote consultation and monitoring.

Telemedicine has already shown some potential benefits in cardiac surgery as a diagnostic tool (ECG, imaging review) for assessing preoperative health status in surgical patients and in managing comorbid conditions in order to reduce surgical risks [28]. Few data are available regarding the implementation of telerehabilitation and they mostly address the remote monitoring of postoperative recovery [29,30]. Nevertheless, these first experiences show that digital health services are associated with improved outcomes [31] and higher patient and operator satisfaction [29,30,31,32], with a high level of patient engagement, especially in the period immediately before and after the surgical procedure [33].

Expanding the treatment for pre-admission and post-discharge phases by offering an asynchronous telemedicine/telerehabilitation service has some advantages for both healthcare providers and patients. No extra outpatient clinic resource is required, which would have involved more staff and new facilities. Furthermore, particularly considering our practice within an academic hospital that serves patients from different surrounding areas and regions, the availability of a remote service would allow patients and relatives to avoid travelling to attend in-person meetings and to follow the education and therapy programmes at their convenience. This can facilitate their compliance while also offering instruments that, in light of the constant increase in the level of digitalization in our country, are widely available and commonly used in everyday life.

Although characterised by a significant heterogeneity of programmes with different delivery modalities and protocols, previous experiences focusing on preoperative rehabilitation found that selected interventions, including breathing exercises, chest physio, aerobic exercise, and muscle strengthening, are associated with improved surgical outcomes and can reduce mortality, pulmonary complications, and ICU stay [34]. Prehabilitation activities appeared beneficial not only for frail patients but also for younger and pauci-symptomatic patients who may adopt sedentary habits and drastically reduce their activity while awaiting cardiac surgery. Furthermore, making patients and their relatives familiar with the hospital environment and the various steps of the surgical journey and providing punctual information regarding expectations and modalities of recovery can improve compliance during the hospitalisation [35,36], decrease stress and anxiety, reduce the incidence of postoperative delirium, and promote recovery [37].

Indeed, surgery has a deep impact on the psychological state as it represents a landmark event in a patient’s life, characterised by uncertainty about the outcome and postoperative recovery [38,39,40]. Most cardiac surgery patients experience significant anxiety in the last few days before the operation and during the time spent on the waiting list. Several studies have reported that anxiety and state depression are associated with a higher risk of complications during the postoperative period [41], including a higher rate of early [41,42] and late mortality [43]. As the stress caused by surgical operations already starts with the diagnosis and the inclusion in the waiting list [40], specialised support is needed for these patients well before hospital admission. Few experiences in cardiac ERAS have proposed integrated actions in preoperative and postoperative psychological assessment and therapy [16]. In our protocol, we have included a comprehensive set of diagnostic tools and the availability of psychological therapy, thus presenting a study of the psychological state of patients and its evolution throughout the surgical journey, the offer of a therapeutic option by our psychiatrists, and the possibility of collecting patient-reported outcomes (PROs) and experience measures (PREMs).

To date, cardiac surgery has focused almost exclusively on meaningful traditional surgical outcomes, including mortality, morbidity, and survival. This view does not fully consider patients’ overall perspective on the health-related quality of life [44], as data on patients’ self-reported physical, mental, and emotional well-being have rarely been reported in experiences including cardiac surgery patients. Furthermore, evidence for using patient-reported experience measures, which can better explore the success of delivering care processes [45], is minimal. In our protocol, the collection of PROs and PREMs will have a dual role, shedding light on patients’ everyday needs and expectations beyond the usual surgical hard endpoints and providing insights into the quality of care during the surgical journey. The use of telemedicine/telerehabilitation seems appropriate for this goal, as it has already been shown to facilitate the collection of patient-reported outcomes and experience data [46] with a high rate of engagement and satisfaction.

Modern cardiac surgery is called upon to satisfy the growing demand for less traumatic treatments, faster recovery, better experience, and postoperative quality of life while maintaining its outstanding consolidated technical results. To respond to these desires and expectations, tailored and person-centred care within a multidisciplinary team is required. Having consolidated our team’s expertise in providing enhanced in-hospital management, we have set up this expanded ERAS pathway, looking at the often unrecognised and unmet needs for psychological and physical support during the pre-admission and post-discharge periods. The use of digital health tools can be welcomed by patients and their relatives and can facilitate the implementation of further therapeutic actions, especially in contexts with a limited availability of human and material resources. Our experience could add more insights into the effectiveness and feasibility of this approach in cardiac surgery patients and, with the collection of high-quality data, could contribute to the production of new evidence necessary to promote the diffusion and support the acceptance of these interventions aimed at the global improvement of patients’ experience throughout the overall surgical journey.

## Data Availability

Data are contained within the article.

## Supplementary Materials

The following supporting information can be downloaded at: https://www.mdpi.com/article/10.3390/jcm14030750/s1, Table S1: The ultra-fast protocol associated with heart valve surgery; Table S2: The TIDieR (Template for Intervention Description and Replication) Checklist; Video S1: Demo of telerehabilitation web-based platform (https://susy.mdpi.com/user/submission/video/2d8904b7617ff6972c54d385c826a9bc).
