# Supplementary material for: Telemedicine/Telerehabilitation to Expand Enhanced Recovery After Surgery Interventions in Minimally Invasive Mitral Valve Surgery
Source: J Clin Med. 2025 Jan 24;14(3):750. doi: 10.3390/jcm14030750 (PMC11818710; doi:10.3390/jcm14030750)
Supplement: Supplementary file 1 [file jcm-14-00750-s001.zip › List of sources of data.pdf]

List of sources of data

TIDieR checklist

- Hoffmann TC, Glasziou PP, Boutron I, Milne R, Perera R, Moher D, Altman DG, Barbour V, Macdonald H, Johnston M, Lamb SE, Dixon-Woods M, McCulloch P, Wyatt JC, Chan AW, Michie S. Better reporting of interventions: template for intervention description and replication (TIDieR) checklist and guide. BMJ. 2014 Mar 7;348:g1687. doi: 10.1136/bmj.g1687

Clinical frailty score and exercise in patients with mitral valve disease

- Rockwood K, Song X, MacKnight C, Bergman H, Hogan DB, McDowell I, Mitnitski A. A global clinical measure of fitness and frailty in elderly people. CMAJ. 2005 Aug 30;173(5):489-95. doi: 10.1503/cmaj.050051
- Perone F, Peruzzi M, Conte E, et al. An Overview of Sport Participation and Exercise Prescription in Mitral Valve Disease. J Cardiovasc Dev Dis. 2023;10(7):304.
- Pelliccia A, Sharma S, Gati S, et al; ESC Scientific Document Group. 2020 ESC Guidelines on sports cardiology and exercise in patients with cardiovascular disease. Eur Heart J. 2021;42:17-96.

Strategy and principles of Telemedicine

- WHO Global Observatory for eHealth. Telemedicine: opportunities and developments in Member States: report on the second global survey on eHealth. World Health Organization. Geneva, 2010. Available from: https://apps.who.int/iris/handle/10665/44497
- World Health Organization. WHO guideline: recommendations on digital interventions for health system strengthening. World Health Organization. Geneva, 2019. Available from: https://apps.who.int/iris/handle/10665/311941. License: CC BY-NC-SA 3.0 IGO
- Global strategy on digital health 2020-2025. Geneva: World Health Organization;2021
- Alexander M. Chapter 1- Introduction. In: Telerehabilitation Principles and Practice. (Ed. Marcalee Alexander). Elsevier, 2022. Available from: https://www.sciencedirect.com/science/article/abs/pii/B9780323824866000010

Internet and mobile consumer trends

- Deloitte. Global Mobile Consumer Trends, 2nd Edition. Deloitte. 2017
- ISTAT. Cittadini e ICT - Anno 2023. 2023
