# Supplementary material for: Telemedicine/Telerehabilitation to Expand Enhanced Recovery After Surgery Interventions in Minimally Invasive Mitral Valve Surgery
Source: J Clin Med. 2025 Jan 24;14(3):750. doi: 10.3390/jcm14030750 (PMC11818710; doi:10.3390/jcm14030750)
Supplement: Supplementary file 1 [file jcm-14-00750-s001.zip › Supplemental Table S1.pdf]

Supplemental Table S1. The ultra-fast protocol associated with heart valve surgery

**Preoperative**	
Risk prevention	- Glycemic control<br>- Correction of nutritional deficiency when feasible<br>- Smoking cessation<br>- Dental and oral hygiene
Nutritional management	- Consumption of clear liquids up until 2 to 4 h before anesthesia<br>- No solid food after midnight
**Intraoperative**	
Reducing surgical site infections	- Depilation protocols<br>- Antibiotic prophylaxis
Surgical access	- Trans-axillary mini-thoracotomy / Mini-sternotomy
CPB management	- Normothermic cardiopulmonary bypass conduct<br>- Reduction of cardiopulmonary bypass fluid (from 12.2021 use of del Nido solution instead of HTK cardioplegia)
Bleeding prevention	- Use of tranexamic acid
Prevention of hypothermia	- Use of forced air warming blankets<br>- Raise the ambient room temperature<br>- Warming irrigation and intravenous fluids
Anaesthesia	- Low-dose opioid-based general anesthesia (5 µg/kg at the induction)<br>- Short-acting volatile agent to maintain anaesthesia<br>- Anterior serratus plane block / Parasternal block<br>- Local anaesthetic infiltration<br>- On table extubation
**Postoperative**	
Pain management	- Continuous analgesia infusion for 12–24 h (Tramadol 4–8 µg/kg/min). For patients with eGFR<50 Tramadol 4 µg/kg/min, a maximal dose of 400mg/day was observed
Temperature control	- Avoidance of persistent hypothermia (<36.0 ◦ C)
Chest tube management	- Maintenance of chest tube patency to prevent retained blood (stripping/milking)<br>- Early drains removal (POD 1) based on drains output, quality of drained fluid and evaluation of chest x ray and bedside echocardiogram
Hemodynamics management	- Goal-directed therapy with fluids, vasopressors and inotropes to avoid hypotension and low cardiac output based on monitoring:<br>o Blood pressure<br>o Cardiac index<br>o Systemic venous oxygen saturation<br>o Urine output<br>o Oxygen consumption<br>o Oxygen debt<br>o Lactates level
Early detection of kidney stress	
Delirium screening	- Systematic delirium screening at least once per nursing shift
Glycemic control	- i.v. Insulin
Thromboprophylaxis	- Pharmacological thromboprophylaxis as soon as satisfactory hemostasis has been achieved
Rehabilitation therapy in ICU	- Respiratory therapy (3–6 hours after extubation)

	- Early mobilization (6–12 hours after extubation):<br>o bed exercises<br>o bed and chair sitting<br>o standing position<br>o ambulation<br>- Oral feeding (6–12 hours after extubation)
Patient-family contact	- Immediate patient-family contact (3 hours after extubation)
ICU discharge	- As early as possible ICU discharge (6–24 hours after extubation)<br>o Patient fully conscious<br>o No inotropes support<br>o No ventilatory support<br>o No uncontrolled arrhythmia<br>o No bleeding<br>o No AKI requiring replacement therapy<br>o No ECG, US and laboratory signs of myocardial ischaemia
**Ward**	
Rehabilitation therapy	- Daily evaluation and treatment for respiratory therapy and mobilization
Delirium screening	- Systematic delirium screening at least once per nursing shift
Glycemic control	- Preoperative therapy & s.c. insulin
Thromboprophylaxis	
Arrhythmia detection	- ECG telemetry till discharge
Correction of anemia	- Transfusion of RBC to be considered for Hb value below 8 g/dl or in symptomatic patients
Home discharge	- As early as possible from POD 5<br>o patient fully mobilized and able to ambulate without any support<br>o stable ECG<br>o satisfactory surgical results and no significant pericardial collection as per pre-discharge echocardiogram performed on POD 4<br>o does not require any cardiac therapeutic input<br>o satisfactory oxygenation with no need of O2 therapy<br>o satisfactory ventilation and absence of significant signs of lung consolidation, pleural collection or pneumothorax as per pre-discharge chest x ray performed on POD 4<br>o normal wound healing<br>o satisfactory values at POD 4 laboratory analysis
